# Regulation of Chemokine and Chemokine Receptor Expression by PPARγ in Adipocytes and Macrophages

**DOI:** 10.1371/journal.pone.0034976

**Published:** 2012-04-17

**Authors:** M. T. Audrey Nguyen, Ai Chen, Wendell J. Lu, WuQiang Fan, Ping-Ping Li, Da Young Oh, David Patsouris

**Affiliations:** Department of Medicine (0673), University of California San Diego, La Jolla, California, United States of America; University Medical Center Freiburg, Germany

## Abstract

**Background:**

PPARγ plays a key role in adipocyte biology, and Rosiglitazone (Rosi), a thiazolidinedione (TZD)/PPARγ agonist, is a potent insulin-sensitizing agent. Recent evidences demonstrate that adipose tissue inflammation links obesity with insulin resistance and that the insulin-sensitizing effects of TZDs result, in part, from their anti-inflammatory properties. However the underlying mechanisms are unclear.

**Methodology and Principal Findings:**

In this study, we establish a link between free fatty acids (FFAs) and PPARγ in the context of obesity-associated inflammation. We show that treatment of adipocytes with FFAs, in particular Arachidonic Acid (ARA), downregulates PPARγ protein and mRNA levels. Furthermore, we demonstrate that the downregulation of PPARγ by ARA requires the activation the of Endoplamsic Reticulum (ER) stress by the TLR4 pathway. Knockdown of adipocyte PPARγ resulted in upregulation of MCP1 gene expression and secretion, leading to enhanced macrophage chemotaxis. Rosi inhibited these effects. In a high fat feeding mouse model, we show that Rosi treatment decreases recruitment of proinflammatory macrophages to epididymal fat. This correlates with decreased chemokine and decreased chemokine receptor expression in adipocytes and macrophages, respectively.

**Conclusions and Significance:**

In summary, we describe a novel link between FAs, the TLR4/ER stress pathway and PPARγ, and adipocyte-driven recruitment of macrophages. We thus both describe an additional potential mechanism for the anti-inflammatory and insulin-sensitizing actions of TZDs and an additional detrimental property associated with the activation of the TLR4 pathway by FA.

## Introduction

Free Fatty Acids (FFAs) are important adipocyte-derived signaling molecules whose plasma levels are elevated in obese and insulin resistant individuals and animal models. FFAs can induce insulin resistance and inflammation in adipocytes, macrophages and skeletal muscle [1], [2], [3], [4], [5], [6]. In 3T3-L1 adipocytes, FFAs decrease adipocyte insulin sensitivity by activating JNK signaling pathways, Endoplasmic Reticulum (ER) stress and increasing secretion of the inflammatory cytokine TNFα [7], [8], [9]. The pro-inflammatory and insulin desensitizing actions of FFAs are mediated by stimulation of Toll-Like Receptor (TLR) 4 in adipocytes and TLR2 and TLR4 in macrophages [4], [5], [9], [10]. Furthermore, FFAs induce the expression of and secretion of numerous chemokines from adipocytes [11], [12]. As these chemokines (e.g. MCP1) potently recruit macrophages, FFAs may indirectly induce insulin resistance, *in vivo*, by stimulating the recruitment of pro-inflammatory leukocytes [13], [14].

PPARγ is a member of the nuclear hormone receptor superfamily involved in numerous developmental and metabolic processes in adipocytes, macrophages, liver, pancreatic beta-cells as well as skeletal muscle [14], [15], [16], [17], [18], [19]. Two isoforms of PPARγ have been described so far in mice, with PPARγ2 expression being restricted to the adipocytes in white adipose tissue [20]. Much of the insight into PPARγ biology has been gained from *in vitro*, *in vivo* and pharmacologic studies using natural and synthetic PPARγ ligands such as 15d-PGJ2 and insulin-sensitizing thiazolidinediones (TZDs) like Rosiglitazone (Rosi) [20], [21], [22], [23], [24]. In peritoneal macrophages, 15d-PGJ2 inhibits gene expression of IP-10 and MCP-1, and treatment with TZDs suppresses the expression and secretion of inflammatory cytokines in monocytes and macrophages [25], [26], [27], [28]. In 3T3L1 mature adipocytes Rosi blocks TNFα- dependent transcriptional effects on multiple glucose/fatty acid metabolism genes as well as adipokines (e.g. adiponectin) [29], [30]. PPARγ-depleted adipocytes also displayed enhanced inflammatory responses to TNF-alpha stimulation, suggesting that PPARγ also plays a role in regulating inflammatory pathways in 3T3-L1 cells [29], [30]. *In vivo*, mice lacking PPARγ in adipose tissue exhibited marked defects in adipocyte morphology, reduced plasma adiponectin levels and elevated plasma FFAs [16]. They were also more susceptible to high fat diet-induced insulin resistance in adipose tissue and liver [16], [31], [32]. When PPARγ expression in macrophages was ablated in mice, this resulted in systemic insulin resistance with attenuated insulin sensitizing effects of TZDs [15]. Recently a novel PPARγ ligand was identified, which possessed weak transactivation but maintained transrepression activity and retained antidiabetic properties [27]. Together these studies suggest that the insulin-sensitizing properties of PPARγ are at least partially due to its anti-inflammatory activity.

In the current studies, our aim was to identify whether there is an interplay between FFAs and PPARγ in the context of obesity-associated inflammation. First, we show that FAs stimulate the secretion of various chemokines from adipocytes into conditioned medium (CM) and that Rosi treatment blocks this effect. Using functional macrophage migration assays, we then demonstrate that the chemotactic properties of CM were correlated to the stimulatory and inhibitory effects of FAs and Rosi, respectively. Furthermore, we present evidence that FAs, Arachidonic Acid (ARA) in particular, decrease PPARγ expression in a TLR4-dependent manner, and that this effect is due to alteration in the transcription of PPARγ. We next investigated the mechanisms involved in this downregulation and establish that ER stress inducers also downregulate PPARγ expression, whereas, ER stress inhibitors prevented the ability of ARA to decrease the expression of PPARγ. Finally, when PPARγ was depleted from adipocytes, the chemotactic activity of CM was enhanced, indicating that chemokine expression is tightly controlled in adipocytes by mechanisms involving negative interactions between PPARγ and the FFAs, TLR4/ER stress pathway.

## Results

### PPARγ signaling decreases secretion of chemoattractants from adipocytes

**Figure 1:**
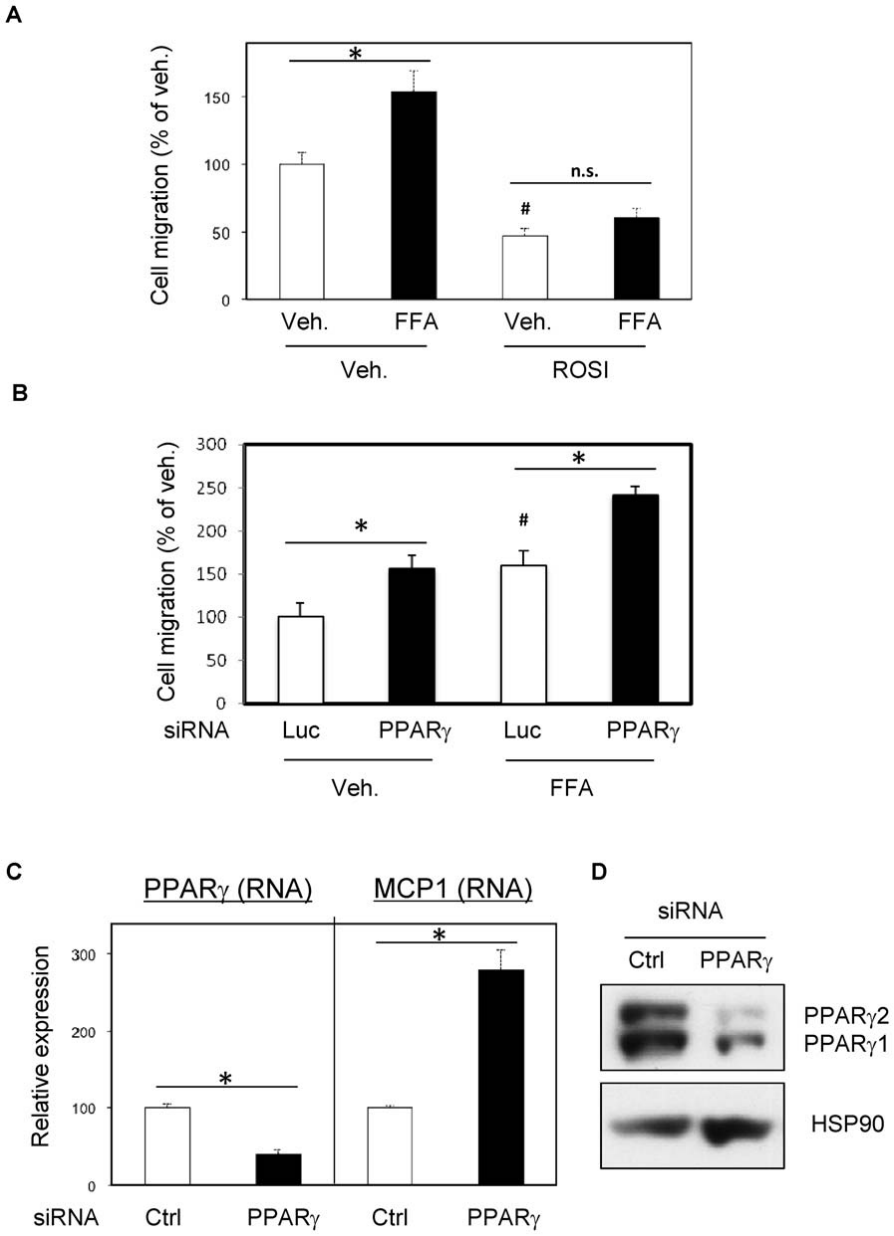
PPARγ signaling decreases secretion of chemoattractants from adipocytes. A) Mature 3T3L1 adipocytes were exposed 24 hours to 500 µM FFA mixture (arachidonic, lauric, linoleic, myristic and oleic acids, 100 µM final each) in presence or absence of Rosi (1 µM). After washes, 24 hours adipocytes conditioned media were prepared, in absence of treatment and used for chemotaxis assay on Raw264.7 macrophages. B) PPARγ siRNA or controls (Ctrl) were injected in mature 3T3L1 adipocytes. 24 hours after electroporation, adipocytes were exposed to FFA mixture or vehicle, for 16 hours. After washes, 24 hours adipocytes conditioned media were prepared, in absence of treatment and used for chemotaxis assay on Raw264.7 macrophages. C) QPCR was used to quantify PPARγ and MCP1 from the adipocytes treated as above. D) Western-blots were performed in order to quantify efficient PPARγ knockdown, HSP90 protein is shown as internal control. * P<0.05; # P<0.05 for Rosi *vs* Vehicle in absence of FFA in 1A; and for PPARγ siRNA *vs* Luciferase siRNA in absence of FFA.

We have previously shown that FFAs elevate chemokine secretion from adipocytes [12]. To determine whether PPARγ activation inhibited this effect, 3T3-L1 adipocytes were treated with FFAs (500 µM final concentrations of arachidonic, lauric, linoleic, myristic and oleic acids, 100 µM final each), in the absence or presence of Rosi (1 µM), followed by measurement of Raw264.7 macrophage chemotaxis in response to adipocyte conditioned media (CM). FFA treatment increased macrophage migration, and this was prevented by Rosi pretreatment (Fig. 1A). SiRNA-induced PPARγ depletion (80% Fig. 1D) enhanced the chemotactic properties of adipocyte CM (Fig. 1B) and also increased MCP1 expression (Fig. 1C).

### FFA treatment decreases adipocyte PPARγ expression

**Figure 2:**
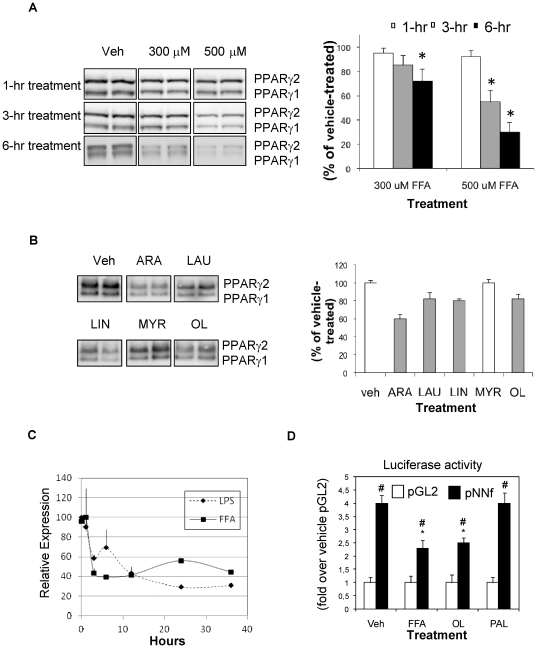
Free Fatty Acid treatment decreases adipocyte PPARγ expression. Differentiated 3T3-L1 adipocytes were treated with 300 µM and 500 µM of a mixture of saturated and unsaturated FFAs for 1–6 hours (A), or with 500 µM of individual FFAs for 3 hours (B) (ARA, arachidonic acid; LAU, lauric acid; LIN, linoleic, acid; MYR, myristic acid; OL, oleic acid). Cell lysates were then analyzed for PPARγ protein content by SDS-PAGE fractionation and immunoblotting using a monoclonal antibody that detects PPARγ1 and PPARγ2 isoforms. Densitometric analysis of PPARγ protein collected from multiple experiments is presented in the right panels (A and B) *P<0.05. C) IPMacs were treated for the indicated times with LPS (5 ng/ml) or FFAs mixture (500 µM) and PPARγ expression was quantified by QPCR. D) 3T3-L1 adipocytes were electroporated with plasmids encompassing parts of the mouse (m)PPARγ2 promoter (PNNf) or no promoter (pGL2) fused to the firefly luciferase gene, along with the beta-galactosidase plasmid. Cells were treated with the FA mixture or with OL and PAL, the two major FAs found in plasma and then lyzed. Luciferase and beta-galactosidase (for normalization) activities were assayed. # P<0.05 for pNNf vs pGL2; * P<0.05 for pNNf of veh-control vs FA treatment.

To better characterize the molecular connections between FA-induced chemotaxis and PPARγ differentiated 3T3-L1 adipocytes were treated with FFAs for 1–6 hours (Fig. 2A) and analyzed for PPARγ protein content by immunoblotting. FFA treatment led to decreased PPARγ protein levels in a time- and concentration-dependent manner, with 70% depletion at 6 hours of incubation. When individual FAs were tested (Fig. 2B), arachidonic (ARA), lauric (LAU), linoleic (LIN), myristic (MYR) and oleic (OL) acids decreased PPARγ protein by ∼40, 18, 20, 0, and 19%, respectively. The down-regulation of PPARγ by FFAs was also observed in primary macrophages (Fig 2C). The effect of LPS (5 ng/ml) is shown for comparison. As illustrated in Figure 2D, transfection of the mPPARγ2 promoter/luciferase construct increased luciferase activity by ∼4 fold (vehicle-treated cells with pGL2 vs pNNf), as expected and previously reported [33]. When adipocytes were treated with FFA or OL, luciferase activity was decreased by ∼33 and 40% respectively, whereas PAL treatment had no effect. This indicated that the FA mixture and OL decrease PPARγ protein levels by decreasing PPARγ promoter activity and gene transcription. We also found that treatment with ARA, LAU, LIN but not MYR can decrease PPARγ promoter activity in 3T3-L1 adipocytes (data not shown).

### Arachidonic Acid prevents PPARγ transrepressional activity on chemokines secretion by adipocytes

**Figure 3:**
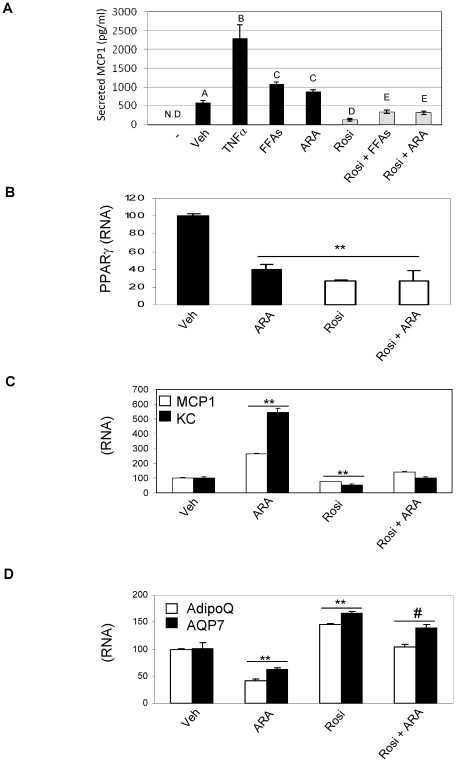
Arachidonic Acid prevents PPARγ transrepressional activity on chemokines secretion by adipocytes. MCP1 concentration was quantified with ELISA from 24 hours 3T3L1 conditioned media (A). Conditioned media were either prepared from adipocytes not stimulated (Veh), treated with TNFα (10 ng/ml), with 500 µM of FFAs mixture (containing 100 µM Arachidonic Acid) or with 100 µM of Arachidonic Acid (ARA) alone. Cotreatment with Rosi (1 µM) was also perfomed (white bars). Media used for preparation of conditioned media is shown for reference (−). QPCR analysis of PPARγ (B) MCP1 and KC (C) and adiponectin (AdipoQ) and aquaporin 7 (AQP7) (D) expression in 3T3L1 adipocytes exposed to 100 µM of Arachidonic Acid (ARA), Rosi (1 µM) or both compounds; ** p<0.01 *vs* Untreated, # p<0.05 *vs* Rosi. Letters above the bars show statistical groups (p<0.05).

As ARA had the greatest effect to down-regulate PPARγ expression (Figure 2B), we assessed the results of ARA treatment on adipocyte chemoattractant secretion. In Figure 3A, we show that 100 µM almost matched the stimulatory effect of 500 µM of the FFA cocktail on MCP1 secretion. Rosi pre-treatment abolished the response to all the pro-inflammatory stimuli tested, i.e. TNFα, FFAs and ARA. Furthermore, secretion of MCP1 by adipocytes paralleled the chemotactic response observed in Fig. 1A. We observed that ARA decreased PPARγ RNA expression by 60% (Figure 3B), an effect that is similar to that known for its agonist, Rosi [34], [35]. We next measured the effect of ARA on either the transrepressional or transactivational activity of PPARγ. As shown, in figure 3 C, ARA treatment did not hamper PPARγ-mediated transrepressional activity on MCP1 or KC expression. In addition, whereas ARA repressed the expression of two known PPARγ target genes, adiponectin (AdipoQ) and aquaporin 7 (AQP7), Rosi still exhibited PPARγ-mediated transactivational activity even in presence of ARA (Figure 3D).

### The downregulation of MCP1 expression by Rosiglitazone does not require de novo protein synthesis

We tested whether the downregulation of chemokine expression by Rosi involved direct PPARγ transrepressional activity or required synthesis of a PPARγ-induced repressor protein. We thus treated adipocytes with TNFα in the presence or absence of the protein synthesis inhibitor, cycloheximide (CHX).

**Figure 4:**
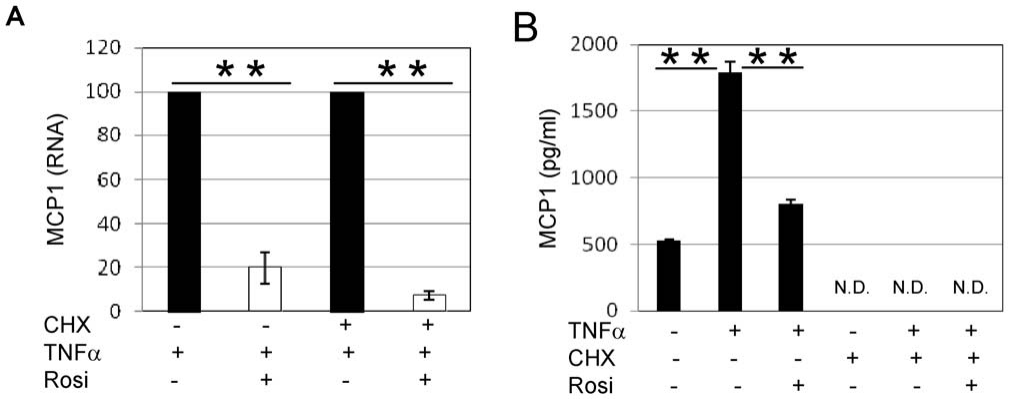
The downregulation of MCP1 expression by Rosi does not require *de novo* protein synthesis. 3T3L1 were treated for 1 Hr with 10 µM cycloheximide (CHX), followed by 1 Hr with Rosi (1 µM) and 5 hours with TNFα (10 ng/ml). MCP1 expression was then quantified with QPCR (A) whereas MCP1 secretion was quantified with ELISA in the corresponding conditioned media (B). ** P<0.01; N.D., Not Detected.

As seen in Figure 4, MCP1 expression was downregulated by Rosi even in the presence of CHX, indicating that Rosi acted in a direct manner (Fig. 4A). In the presence of CHX, MCP1 was not detected in adipocyte CM, demonstrating the efficacy of CHX in these experiments (Fig. 4B).

### The downregulation of PPARγ expression by FFAs requires TLR4 dependent activation of the ER stress

**Figure 5:**
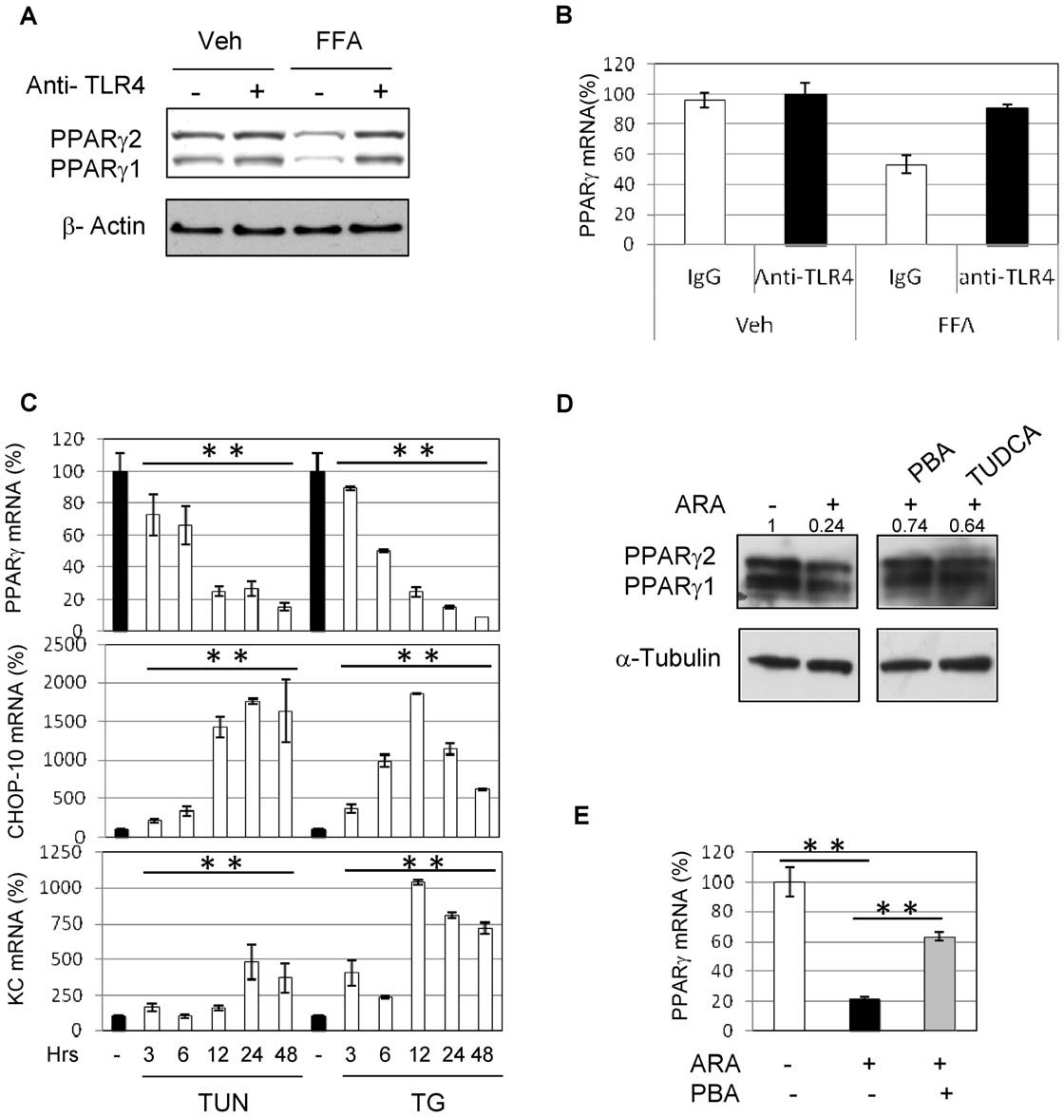
The downregulation of PPARγ expression by FFAs requires TLR4dependent activation of the ER stress. 3T3L1 adipocytes were pretreated with neutralizing antibodies to TLR4 (A, B), or control IgG prior to FA treatment (500 µM, 3 hr). Adipocyte PPARγ protein content was assessed as above, by immunoblotting analysis (A) and mRNA by real time qPCR (B). C) Mature 3T3L1 were treated with tunicamycin (TUN, 1 µg/ml) and thapsigargin (TG, 50 nM) for the indicated times prior to extract RNA and quantify PPARγ, CHOP-10 and KC with qPCR. D) Mature 3T3L1 adipocytes were pretreated 3 hours with 14-phenyl butyrate (PBA, 10 mM) and tauroursodeoxycholic acid (TUDCA, 500 µg/ml) prior to be exposed to ARA (100 µM) for 6 hours. Proteins were then extracted and PPARγ was quantified by western-blot. E) Mature 3T3L1 adipocytes were pretreated 3 hours with 14-phenyl butyrate (PBA, 10 mM) before exposure to 100 µM ARA. PPARγ expression was quantified with qPCR. ** P<0.01.

To determine the mechanisms involved in FFA-induced downregulation of PPARγ expression we pretreated adipocytes with TLR4 (5A, 5B) or TNFα (not shown) neutralizing antibodies prior to treatment with FFAs (500 µM, 3 hours). Whereas neutralizing TNFα did not alter the ability of FFAs to decrease PPARγ expression, neutralizing TLR4 prevented this effect at the protein (5A) and gene expression level (5B). As ER stress is a known side effects associated with FAs exposure and TLR activation, we next tested whether ER stress inducers would mimic the detrimental effects of FAs treatments [8], [36]. As shown in figure 5 C, we indeed observed that similar to ARA, the ER stress inducers, thapsigargin (TG, 50 nM) and tunicamycin (TUN, 1 µg/ml), strongly decreased the expression of PPARγ (−80% and −90% respectively). In addition, the ER stress response gene CHOP-10 was upregulated with the downregulation of PPARγ. This was also correlated with the increased expression of the chemokine KC. We consequently tested whether ER stress inhibitors could interfere with the ability of ARA to downregulate the expression of PPARγ in mature 3T3L1 adipocytes. We found that treatment with the two chemical chaperones, 4-phenyl butyrate (PBA, 10 mM)) and tauroursodeoxycholic acid (TUDCA, 500 µg/ml), strongly prevented the downregulation of ARA-induced PPARγ expression. Six hours of treatment with ARA (100 µM) decreased PPARγ protein level to 24% compared to untreated cells. PBA and TUDCA treatment maintained PPARγ expression to a relative level of 74% and 64% respectively (Figure 5D). This effect was also seen at the transcriptional level (PPARγ RNA, fig. 5E). In addition, we looked at other mediators of TLR4 biological actions. Consequently, we knocked down IRF3, TAK1 and JNK and observed that these manipulations did not interefere with the ability of FAs to decrease PPARγ expression (not shown) [8], [37], [38]. Lastly, overexpression of a super-repressor of NFκB (DN-IκB) also had no effect (not shown).

### Chronic treatment with Rosiglitazone causes increased adiposity with decreased Adipose Tissue Macrophage (ATM) content

**Figure 6:**
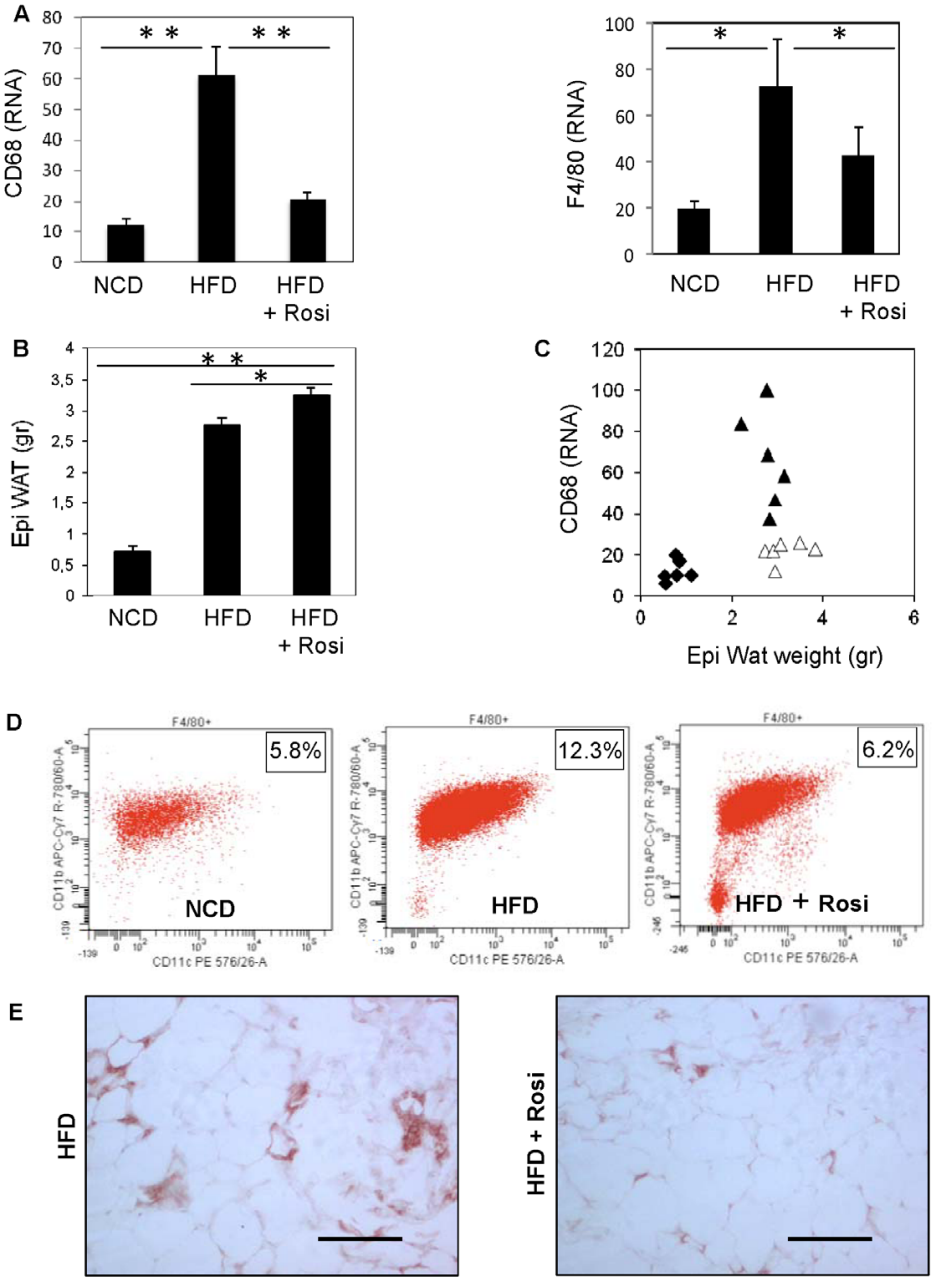
Chronic treatment with Rosi causes increased adiposity with decreased ATM content. A) QPCR quantification of macrophages markers, CD68 and F4/80, in epidydimal WAT of mice fed 16 weeks with a normal chow diet (NCD), a high fat diet (HFD) in absence or presence of Rosi. Epididymal fat weights of mice described above (B) and association between CD68 expression and eWAT (C) * P<0.05; ** P<0.01. D) Facs analysis of F4/80, CD11b and CD11c were performed on cells from stromal vascular fractions of mice fed with normal chow, high fat diet (16 weeks) or high fat diet for 13 weeks followed by three weeks treatment with Rosi. Plots data represent the relative expression of CD11b and CD11c in F4/80 gated cells. E) Immunostaining of F4/80 on eWAT section of mice fed a high fat diet alone (16 weeks) or in presence of Rosi for the last three weeks.

To determine whether our *in vitro* findings on chemokine secretion and macrophage chemotaxis translate to the *in vivo* situation, we quantified macrophage markers in epididymal fat of mice fed either normal chow or high fat diet (HFD) for 16 weeks in the presence or absence of Rosi. Both CD68 and F4/80 (Fig. 6A) expression were increased by HFD, which indicated an increased presence of ATMs. Expression of these markers was strikingly diminished upon Rosi treatment. Feeding with HFD was also associated with an increase in epididymal fat weight, which was slightly more pronounced with Rosi supplementation (Fig. 6B). Thus, the correlation between adiposity and ATMs was dissociated with Rosi treatment (Fig. 6C).

We and others have shown that the proinflammatory macrophages which accumulate in adipose tissue express high levels of CD11c [39], [40]. Thus, we prepared Stromal Vascular Cells (SVCs), the macrophage containing cellular fraction, from adipose tissue for FACS analysis. The FACS results show that HFD increased the number of F4/80+, CD11b+, CD11c+ ATMs, which was normalized by Rosi treatment (fig. 6D). Decreased ATMs recruitment was confirmed with adipose tissue immunohistochemistry staining of F4/80+ cells (Fig. 6E). Interestingly, decreased ATMs content was only observed after three weeks treatment with rosiglitazone and no difference were observed after one week treatment with Rosi. However, *in vivo* tracking experiments with labeled macrophages demonstrate that ATMs are long lived cells as injected macrophages are still detectable 28 days after injection [41]. Consequently one could porpose that even though rosiglitazone may quickly lead to a decreased expression and secretion of chemokines, this may not immediately translate in a decrease of ATMs.

### Rosiglitazone decreases chemokine expression in adipocytes and chemokine receptor expression in SVCs

**Figure 7:**
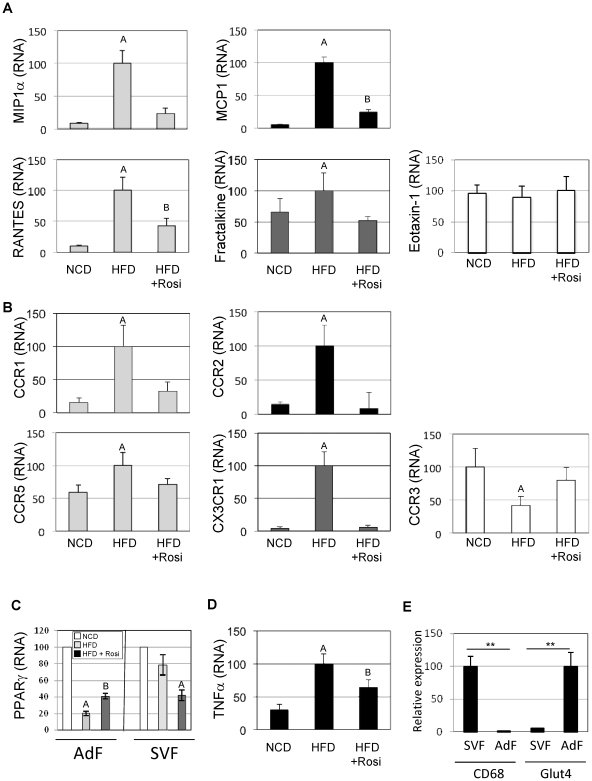
Rosiglitazone decreases chemokine expression in adipocytes and chemokine receptor expression in SVCs. Expression of macrophages chemokines in adipocytes fraction (A) and corresponding receptors (B) in stromal vascular fraction were quantified with QPCR. Eotaxin-1 and corresponding receptor, CCR3 are shown in white, MIP1α and RANTES corresponding receptors CCR1 and CCR5 are shown in light grey, Fractalkine and corresponding receptor CX3CR1 are shown in dark grey, MCP1 and corresponding receptor, CCR2, are shown in black. C) QPCR analysis of PPARγ in adipocytes and SV fractions. D) TNFα expression in stromal vascular fraction was determined by qPCR. D) CD68 and Glut4 expression were quantified from SVF and adipocytes fractions by qPCR. ** P<0.01. A, B above the bars show statistical groups (p<0.05).

To better understand the mechanism of macrophage recruitment to adipose tissue and the effects of Rosi on this process, we measured mRNA expression of known chemokines in adipocyte fractions prepared from epididymal fat pads (Fig. 7A). HFD led to increased expression of all the measured adipocyte-derived chemokines; i.e., fractalkine (CX3CL1), MCP-1 (CCL2), MIP1α (CCL3), and RANTES (CCL5). In contrast, the expression of Eotaxin-1 (CCL11), an eosinophil specific chemokine [42], did not change. We also prepared SVCs for analysis. As seen in Fig. 7B, HFD led to increased expression of all the corresponding chemokine receptors. Rosi treatment had broad effects to decrease the HFD-induced increase in chemokine and chemokine receptor gene expression.

In figure 7C, we observed an interesting parallel between the expression of chemokines reported in 7A and PPARγ in adipocytes fraction. Indeed, in high fat diet conditions, the downregulation of PPARγ was associated with the increased expression of the chemokines, whereas treatment with Rosi restored PPARγ expression, while decreasing the expression of chemokines.

Adipose tissue TNFα expression was also increased by HFD and repressed by Rosi treatment Fig. 7D). Since TNFα can stimulate chemokine secretion [12], [43], this observation raises the possibility that Rosi affects chemokine expression by down regulating TNFα.

CD68 and GLUT4 are markers for macrophages and adipocytes, respectively, and Fig. 7E indicates minimal cross contamination between these fractions.

## Discussion

In this study, we show that *in vitro*, FFA treatment of fat cells, particularly ARA, leads to an increase in chemokine expression and secretion from these cells and enhances macrophage migration in chemotaxis assays. These effects are most likely due to the actions of FFAs to activate proinflammatory pathways within adipocytes, which, would in turn stimulate chemokine gene expression and secretion [5], [9], [12], [44]. We further present evidence linking the actions of FFAs to decreased PPARγ activity. We report that FFA treatment led to downregulation of PPARγ expression both in adipocytes and macrophages and that siRNA-mediated knockdown of PPARγ resulted in increased chemokine secretion from adipocytes as well as increased functional chemotaxic activity of CM harvested from PPARγ -depleted adipocytes. These results are likely of physiological importance as they suggest that FFAs exert their stimulatory effects, at least in part, by causing decreased PPARγ expression both in adipocytes and macrophages. Rosi treatment reverses all of these FFA effects on chemokines/chemokine receptors, which further reinforces the important role of PPARγ signaling in this mechanism. As PPARγ signaling normally serves to keep chemokine and chemokine receptor expression low, our results provide a mechanistic explanation as to the pathway whereby FFAs induce chemokine/chemokine receptor expression.

These findings translated to the *in vivo* situation. Our animal data show that HFD led to increased expression of chemokines in mouse tissue adipocytes along with a corresponding increase in chemokine receptors in stromal vascular cell fractions from epididymal fat pads. Recent work indicates the existence of a feed forward pathway between FAs released from adipocytes and cytokine secretion by macrophages, in which FFAs can activate proinflammatory pathways in macrophages and cytokines released from macrophages can stimulate lipolysis in adipocytes [43]. The FAs also lead to proinflammatory effects in adipocytes causing increased chemokine secretion. In addition, FFAs also led to decreased PPARγ expression in macrophages, contributing to their proinflammatory effects in this cell type. Together our results further support that PPARγ plays an important role in integrating communication between the adipocyte and macrophage cell types in the overall chemotactic response of immune cells to chemokines released by adipocytes.

Even though, it is well established that chemokine secretion govern ATMs recruitment *in vivo*, the exact nature of the initial stimuli that drives this program is a subject of intense debate. Several stimuli upstream of chemokines expression and secretion have been proposed so far, including adipocyte interactions with the extracellular matrix, hypoxia and ER stress [45] [13]. Our findings support the idea that ER stress is an essential component in the recruitment of macrophages in response to an obesogenic environment. In agreement with our observations, Jiao et al described that FFA induce ER stress, which further cause insulin resistance in adipocytes [7]. In our manuscript, we bring forward evidences that FFAs may cause ER stress, which decreases PPARγ expression in adipocytes. The exact mechanisms involved in this regulation are still unclear. However, it is known that CHOP-10 displays transrepressional activity on several genes, which further worsens the consequences of ER stress [8], [46]. In particular, it was demonstrated that CHOP-10 decreases the expression of PPARγ in a mechanism involving the sequestration of C/EBPβ and limiting its binding to the PPARγ promoter [47]. The mechanism proposed in this publication perfectly fits with our observations as PPARγ expression is regulated by C/EBPβ binding in adipocytes [48]. Further illustrating the contribution of the TLR4 pathway to this regulation, it is acknowledged that TLR4 activation by LPS decreases PPARγ expression in mature adipocytes [49]. However, future work should be conducted to fully establish that the ER stress is necessary to the TLR4 pathway in order to downregulate the expression of PPARγ in response to FFA.

Our studies further show that Rosi blocked the effects of HFD on chemokine/chemokine receptor expression. These molecular events reflected the state of ATMs accumulation: HFD led to increased ATM content which was completely reversed by three weeks of Rosi treatment. Interestingly, we and others observed that PPAR activators downregulate the expression of PPARγ in 3T3L1 adipocytes [34], [35], [50], and herein report that Rosi, a potent PPARγ agonist, also decreases PPARγ expression. It has been surmised that in adipocytes the downregulation of PPARγ induced upon binding of an agonist most likely describes an auto-regulation process [34]. As illustrated through the expression of PPARγ target genes, the presence of Rosi overwhelms the downregulation induced by both its exogenous agonist and FAs. This could explain why Rosi is observed to be still fully active in preventing macrophages infiltration *in vivo* and *in vitro* despite such a reduction in receptor availability.

In macrophages, the anti-inflammatory properties of TZDs largely rely on PPARγ's ability to decrease the expression of pro-inflammatory cytokines and chemokines, i.e. PPARγ transrepressional activity. In adipocytes, the adipogenic/transactivational activity of PPARγ is well established; however, very little is known about PPARγ's anti-inflammatory/transrepressional properties in adipocytes. Our manuscript establishes how elevated FAs, as would be observed in the context of obesity, decrease PPARγ expression, which further prevents PPARγ transrepressional activity, further resulting in the upregulation of macrophage chemoattractant secretion by adipocytes. This loop of interactions between fatty acids and PPARγ is of particular interest since a recent publication demonstrates that beneficial properties of TZDs may widely rely on PPARγ transrepressional/anti-inflammatory properties [27].

Arachidonic acid and its metabolites are known to activate inflammatory responses in a variety of immune cells [51]. However, in adipocytes, the effects of ARA are poorly documented. We found that ARA was uniquely potent in downregulating PPARγ expression and in stimulating the ability of adipocyte CM to promote chemotaxis. Interestingly, a recent publication reports that deletion of 12/15-lipoxygenase (an enzyme that produces metabolites from ARA) in mice leads to protection from HFD-induced ATM recruitment and inflammation [52]. Thus, the ARA stimulation of chemokine expression in response to adipocytes could involve elevated production of 12/15-lipoxygenase products from ARA.

In summary, our studies show that FFAs decrease PPARγ protein and mRNA levels by decreasing PPARγ gene transcription. This results in increased MCP1 gene expression and enhanced adipocyte-induced monocyte/macrophage chemotaxis. Together our findings indicate that high FFA and low PPARγ levels could enhance the recruitment of inflammatory macrophages into adipose tissue, contributing to the development of tissue inflammation and insulin resistance. Moreover, TZD treatment may decrease inflammation and improve insulin sensitivity, at least in part, by decreasing ATM recruitment through transrepressional mechanisms directly within adipocytes.

## Materials and Methods

### Ethics Statement

Mouse procedures conformed to the Guide for Care and Use of Laboratory Animals of the US National Institutes of Health, and were approved by the Animal Subjects Committee of the University of California, San Diego, CA, USA (UCSD protocol # S99173).

### Chemicals and Reagents

Cycloheximide, lipopolysaccharide (LPS), free fatty acids, thapsigargin, arachidonic, lauric, linoleic, myristic and oleic acids were from Sigma. Tunicamycin, 14-phenyl butyrate and tauroursodeoxycholic acid were from Calbiochem. Rosiglitazone was from Alexis. Antibodies to PPARγ and horseradish peroxidase (HRP)-linked secondary antibodies were from Santa Cruz Biotechnology (Santa Cruz, CA). Dubbelco's Modified Eagle Medium (DMEM) and fetal bovine serum were purchased from Invitrogen (Carlsbad, CA). TNFα was from Biosource. Low endotoxin, fatty acids free-BSA was from MP Biochemicals (Aurora, OH). PPARγ promoter-luciferase reporter plasmid was kindly gifted by Dr Reddy JK [33].

### Cell culture

Mouse 3T3L1 cells were grown and maintained undifferentiated in DMEM (25 mM glucose) supplemented with 10% Bovine Calf Serum (Hyclone) and differentiated to 3T3L1 adipocytes as previously described (14). Raw264.7 cells were maintained in 10%, low endotoxin FBS (Hyclone) DMEM (25 mM glucose). Unless indicated otherwise, adipocytes were used 10 days after differentiation. Cells were serum-starved in DMEM supplemented with FA-free bovine albumin and treated with an FFA cocktail composed of lauric, myristic, linoleic, oleic and arachidonic acids (1∶1∶1∶1∶1) or individual FAs (lauric, myristic, linoleic, oleic, arachidonic and palmitic; Sigma-Aldrich) or ethanol/PBS vehicle for indicated amounts of time at 37°C. All FA solutions were pre-equilibrated with BSA at 37°C for 1.5 hour and a 5∶1 (mol/mol) FA∶BSA ratio was used to simulate elevated FFAs levels [3]. 3T3L1 and Raw 264.7 cells were obtained from ATCC (American Type Culture Collection, Manassas, VA).

### SiRNA electroporation

SiRNAs were obtained from IDT. Sequences for PPARγ duplexes were as described by Liao [29]. Adipocytes at day +6 from differentiation protocol were collected and electroporated with siRNAs as previsouly described (GENE PULSER; Bio-Rad) [29].

### Neutralizing TLR4 signaling

Prior to the addition of FFAs, each well in a 12-well culture plate was supplemented with Toll- Like Receptor 4 (TLR4) (e-Bioscience, San Diego, CA) neutralizing antibody or control Armenian IgG (10 µg/ml final concentration).

### *In vitro* chemotaxis assay

Following incubation with FFAs or vehicle, 3T3-L1 adipocytes were rinsed with warm DMEM to remove treatments. Cells were subsequently incubated in DMEM containing 0.2% BSA for 24 hours. Conditioned medium (CM) was collected and kept frozen in single-use aliquots. For the chemotaxis assay, 600 µl of adipocyte CM was aliquoted per well of 24-well tissue culture plates, and 200.000 RAW264.7 macrophage cells resuspended in DMEM containing 0.2% of endotoxin- and FFA-free BSA were plated in the upper transwell chamber (8 µm, 24-transwell format; Corning, Lowell, MA). After 3 hours of migration, Raw264.7 cells were fixed in formaldehyde and stained with DAPI. Cells in the upper chamber that had not migrated were removed. RAW264.7 cells found on the filter facing the lower chamber were counted as cells having performed chemotaxis and quantified with the Simple PCI imaging software (Compix Inc., Cranberry Township, PA) [12].

### Animal experiments

Male C57BL/6J mice (littermates) were obtained from Jackson Laboratories and housed four per cage under pathogen free conditions. 10 weeks old mice were fed a high fat diet (60% Kcal fat, Research Diet) where Rosi was directly mixed by manufacturer. Normal chow diet consisted of 13,5% kcal fat (Lab Diet). In figure 5A, 5B and 5C, mice were fed the different diets for 16 weeks. In figure 5D and 5E, mice were fed a normal chow or a high fat diet for 16 weeks that included Rosi for the last three weeks in the designated group.

### Immunohistochemistry

Adipose (epididymal) tissue was fixed in paraformaldehyde (10%) for 24 hours prior to paraffin imbedding. F4/80 (serotec) staining was performed as described previously [40]. Brightfield photographs were taken of 5 representative fields per slide in a blinded fashion using a fluorescent microscope (10× objective).

### Epididymal and adipocytes isolation and FACS analysis of macrophages

Detailed experimental procedures are described by Inouye [53]. In brief, epididymal fat pads were harvested and minced in FACS buffer. Tissues were treated with collagenase for 30 min at 37 C. After treatment, the suspension was filtered and centrifugated. The adipocyte fraction consisted of the supernatant. The SVF pellets were incubated with red blood cell lysis buffer and directly used for RNA isolation or resuspended in FACS buffer. The stromal vascular cells (SVC) were incubated with blocker followed by primary and secondary antibodies with washes in between. SVC were analyzed using FACSCalibur and FACSAria flow cytometers.

### RNA isolation and quantitative PCR

Total RNA was extracted from cells or tissue with Trizol (Invitrogen) reagent. Total RNA was reverse-transcribed with using ABI cDNA Synthesis Kit (Applied biosciences). Primer sequences used in the PCR reactions were chosen based on the sequences available in GenBank and were designed to generate a PCR amplification product of 100–200 bp. Only primer pairs yielding unique amplification products without primer dimer formation were subsequently used. Primer sequences are available upon request. PCR was performed as described [40]. The mRNA expression of all genes reported is normalized to the cyclophilin A gene expression.

### Protein isolation and Western blots

Proteins from tissues or cell lysates were extracted with RIPA buffer (Upstate) in presence of phosphatase- and protease-inhibitors (Roche). 20 µg of proteins per lane were separated on 10% polyacrylamide, precasted SDS gel (biorad) followed by a transfer on PVDF membrane (Immobilon, Millipore) and immunoblotting was performed as described [29].

### ELISA assays

MCP1 was directly quantified from media according to manufacturers instructions (Biosource).

### Statistical analysis

Data represent the means ± S.D. and were evaluated by Student's two-tailed t-test or analysis of variance provided by VassarStats: Web Site for Statistical Computation. A *p* value cut-off of 0.05 was used to determine significance.
